# Association of Endothelial Function with Parental Hypertension in Normotensive-Obese African-American Women: A Pilot Study

**DOI:** 10.1155/2019/5854219

**Published:** 2019-02-03

**Authors:** Vernon Bond, Karissa Becknel, Krishna Kumar, James Dorsey, Vasavi R. Gorantla, Yulia A. Volkova, Richard M. Millis

**Affiliations:** ^1^Department of Recreation, Human Performance & Leisure Studies and Exercise Science & Human Nutrition Laboratory, Howard University Cancer Center, Washington DC, USA; ^2^Department of Pharmaceutical Sciences, Howard University College of Pharmacy, Washington DC, USA; ^3^Departments of Basic Sciences and Clinical Medicine, American University of Antigua College of Medicine, Antigua and Barbuda

## Abstract

Obese African-American (AA) women are at high risk of hypertension (HT) and cardiovascular disease (CVD). Flow-mediated dilation (FMD) and arterial augmentation index (AI) are measures of endothelial function and arterial stiffness. Whether endothelial function and arterial stiffness predict risk of HT or CVD in obese African-American women with, versus without, parental histories of HT and whether aerobic exercise is an effective countermeasure remain unclear. The capacity for FMD is partly heritable. Therefore, we tested the hypotheses that less FMD and greater AI may be found in normotensive-obese, young-adult (18-26 year-old) AA women with hypertensive parents (n=10) than in a matched control group with normotensive parents (n=10) and that a single bout of aerobic exercise improves both endothelial function and arterial stiffness, with less improvement in the women with hypertensive parents. We studied each subject while at rest, 20 min before and 20 min after, 30 min of aerobic exercise. The exercise-induced changes and parental hypertension-related differences in AI were not significant. The exercise increased FMD in both of the groups with no significant difference in magnitude between the women with hypertensive and normotensive parents. FMD was significantly less in the women with hypertensive parents than in the women with normotensive parents after, but not before, the exercise (mean ±95% confidence interval of 11.3 ± 4.9% vs. 15.6 ± 4.9%, P=0.05). These findings suggest that a 30-min bout of aerobic exercise may improve FMD and unmask endothelial dysfunction in normotensive-obese, young-adult AA women with parental histories of HT. Future studies should determine whether regular aerobic exercise protects obese AA women from the endothelial dysfunction associated with diabetes and prevents CVD in this high-risk population.

## 1. Introduction

Arterial pulse pressure, augmentation index (AI), and flow-mediated dilation (FMD) are well-established indicators of arterial stiffness and endothelial dysfunction [1]. These measures of vascular structure and function are also purported to be useful markers for and early predictors of hypertension, atherosclerosis, and cardiovascular disease (CVD) [2, 3]. Vascular health is affected by many factors such as obesity, parental hypertension, ethnicity, and physical activity. Prior studies examining the relationship between ethnicity and vascular function have demonstrated that Americans of African descent have impaired endothelium-dependent and -independent vasodilatation compared to Americans of European descent (Caucasians) [4, 5]. In that regard, young African-American (AA) subjects free from prevalent CVD appear to exhibit a higher prevalence of abnormal blood pressure wave reflections, arterial wall thickness, and arterial stiffness than Caucasians [6].

The prevalence of obesity has increased rapidly over the past decade, with the largest increase in AA women [7]. By 2020, 70% of AA women are projected to be obese [8]. Endothelial dysfunction and arterial stiffness are thought to be the earliest manifestations of impaired vascular function in obesity and precede the development of prehypertension and hypertension [9–11]. When matched for age, pulse wave velocity, a key measure of arterial stiffness, is reported to be ~0.5 m/s higher in obese than in nonobese individuals [12].

The heritability of both arterial stiffness [13–15] and hypertension [16–20] is demonstrated; indeed, one of the highest incidences of hypertension is reported in African Americans [21]. Pulse wave velocity and AI and markers of arterial stiffness were found, in the Framingham Heart Study, to be significant indicators of normotensive individuals with parental histories of hypertension [22].

Physical activity induces chemical and mechanical signals which activate numerous biochemical pathways promoting cardiovascular health [23, 24]. Exercise, even for a brief period, has a prominent effect on vascular endothelial function by improving vasodilator-to-vasoconstrictor balance, reducing inflammation and oxidative stress, and increasing growth of capillaries in skeletal muscles [25–27].

These findings suggest that arterial stiffness and endothelial dysfunction are key factors in the pathogenesis and heritability of clinical hypertension. This pilot study was, therefore, designed to measure radial and aortic pulse pressures, arterial AI, and brachial FMD before and immediately after aerobic exercise.

## 2. Methods

This study tests the hypothesis that normotensive-obese young-adult AA women with hypertensive parents exhibit greater arterial stiffness and less endothelium-dependent vasodilation than a matched group with normotensive parents and that a single bout of aerobic exercise improves both endothelial function and arterial stiffness.

Obese young-adult AA females (age 18-26 years), with and without parental histories of hypertension, were recruited and completed this study (N=20, 10/group). All participants provided written informed consent as approved by the Institutional Review Board (IRB) of Howard University. Exclusion criteria were as follows: histories of diabetes mellitus, hyperlipidemia, hypertension, current smoking, ingestion of caffeine within the past 12 hours before reporting to the laboratory for testing, or any acute medical problem. Obesity was defined by the National Institutes of Health cutoff point as a woman's percentage of body fat >30% [28], using a dual-energy X-ray absorptiometry (DEXA) scan for this measurement. Family histories of hypertension were defined as at least one parent with essential hypertension, confirmed by the presence of ongoing pharmacologic therapy. Parental blood pressure status was confirmed by a research assistant's presence when each of the subjects contacted their parents by telephone and inquired about their blood pressure.

### 2.1. Total Body Fat

Among the body composition techniques, DEXA is accepted as the most suitable method to measure body composition with high accuracy [29, 30]. Body composition was analyzed by means of a Hologic Discovery QDR Series DEXA scanner, with Advanced Body Composition™ assessment (Hologic, Inc., Madison, WI.). The scanner was calibrated daily according to the manufacturer's instruction using a standardized block.

### 2.2. Peak Oxygen Uptake

Participants performed a progressive exercise test on an electric-brake Lode Corival cycling ergometer (Lode, Groningen, the Netherlands) to determine peak oxygen uptake (VO_2peak_). The progressive exercise test involved a 5-min warm-up at 20 W followed by a continuous incremental progression in power of 20 W every 3 min to the limit of volitional fatigue. Expired air was analyzed via an online breath-by-breath system (Max II, Physio-Dyne Instrument, Quogue, NY). Minute ventilation volume was inferred from the measurement of gas flow using a digital turbine transducer. Oxygen analyzer and carbon dioxide analyzer were calibrated with known medical grade gases before each test. VO_2peak_ was defined as the VO_2_ value measured during the last 60 s of work.

### 2.3. Arterial Stiffness

Arterial stiffness was measured using pulse wave analysis evaluating AI at the radial artery. AI is defined as the ratio of augmentation pressure (difference in pressure between the early and late systolic shoulders) to pulse pressure [31]. AI was measured using a noninvasive device, the SphygmoCor (AtCor Medical, Sydney, Australia) which allows online pulse wave analysis recording and automatic calculation [32]. The SphygmorCor system synthesizes a central (ascending aortic) pressure wave from the radial pressure waveform, reported to be equivalent to an intra-arterially recorded waveform [33] using a generalized transfer function [34]. Al was measured at the left wrist with the SphymorCor applanation tonometry. All measurements were made in triplicate, and mean values were used for subsequent analysis.

### 2.4. Endothelial Function

Endothelial function was evaluated by FMD in the brachial artery using a high-frequency CV70 ultrasound (Siemens Medical Solutions, Inc., Mountain View, CA) in accordance with published guidelines [35]. A blood pressure cuff was placed on the left upper arm before imaging. Using a 10 MHz linear array vascular ultrasound transducer and CV70 ultrasound system, the brachial artery was located above the elbow and scanned in longitudinal sections. After recording baseline B-moded images of the brachial artery and spectral Doppler images of flow, the cuff was inflated to 250 mmHg for 5 min to induce reactive hyperemia. Immediately after cuff deflation, spectral Doppler images were obtained to verify hyperemia. Brachial artery images were obtained 40 and 90 s later. The outcome variable of interest was maximum FMD (%), the largest percentage change in brachial artery diameter after reactive hyperemia relative to the baseline diameter.

## 3. Procedures

All participants entered the laboratory with prior instructions limiting food intake and exercise for 4 h. Initially, the participant was positioned supine and body composition was analyzed by the DEXA scan. The participant was instrumented with the SunTech 4240 automated blood pressure device (SunTech 4240, SunTech Medical, Morrisville, NC) and metabolic cart, and then performed the progressive exercise test determining VO_2peak_. Heart rate, blood pressure, and VO_2_ were measured continuously during the progressive exercise test. Approximately 1-3 weeks after determining VO_2peak_, participants entered the laboratory under conditions similar to that of the progressive exercise test of VO_2peak_. Measures of AI and FMD were determined before and after steady-state submaximal exercise. Subjects were placed in a supine position for 10 min before the Al measurement. Brachial systolic pressure and diastolic pressure were measured using the SunTech automated device. At least four brachial blood pressure measurements were taken within a 5-min interval until the differences between systolic pressures were <10 mmHg; the average of the last two blood pressure readings was used to determine the blood pressure parameters for the pulse wave analysis. The subjects performed the exercise protocol consisting of cycling for 30 min on the cycle ergometer at 60% of their predetermined VO_2peak_. Al and FMD were measured 20 min after the 30 min period of aerobic exercise.

## 4. Statistical Analysis

All variables were tested for a normal distribution of the data. Normally distributed data were expressed as means ± 95% confidence intervals. Differences for between and within groups were evaluated using ANOVA with post-hoc t-testing. Equality of variances was verified using the F-test for equal variances and the computed F-statistics were found to be less than the critical values of F. Correlations were found using Pearson's product-moment coefficient. Pre-postexercise percent changes were used to determine whether exercise improved FMD more in the comparison group with family histories of hypertension than in the control group without family histories of hypertension (one-tailed t-test for means with equal variances). P ≤ 0.05 was considered statistically significant. GraphPad Instat software (GraphPad Software, La Jolla, CA) was used for statistical analyses.

## 5. Results

### 5.1. Group Characteristics

The cohort consists of normotensive-obese, young-adult African-American (AA) women, with and without parental histories of hypertension. The two groups did not differ with respect to age (computed as means ± standard errors 21.1 ± 0.3 vs 21.2 ± 0.6 years), height (165 ± 1.9 vs 165 cm), weight (84.8 ± 3.2 vs 79.3 ± 3.8 kg), fat mass (37.4 ± 1.4 vs 36.0 ± 1.0%), systolic pressure (108.6 ± 3.3 vs 112.6 ± 3.1 mm Hg), diastolic pressure (76.6 ± 2.9 vs 76.0 ± 2.2 mm Hg), or VO_2peak_ (19.2 ± 0.9 vs 21.6 ± 1.0 L·min^**-1**^·kg^**-1**^).

### 5.2. Hemodynamics and Pule Wave Analysis

Table 1 shows that arterial augmentation index (AI), heart rate, brachial and central aortic systolic, and diastolic and pulse pressures were not different between the two study groups before or after an exercise treatment. The pre-exercise baseline AI was negatively correlated with body height (r = -0.47, P<0.01).

### 5.3. Endothelial Function

**Figure 1:**
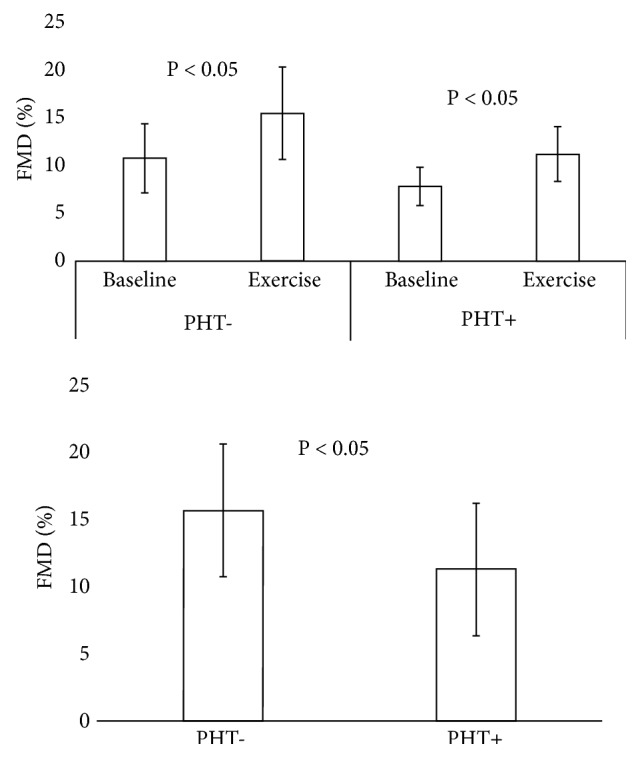
Effect of parental histories of hypertension on flow-mediated dilation (FMD) before and after an exercise treatment. Bars show the means ± 95% confidence intervals of percent changes in brachial artery blood flow during reactive hyperemia induced by blood flow occlusion. These FMD measurements were made 20 min before and 20 min after 30 min of aerobic exercise at 60% of predetermined peak oxygen consumption in matched groups of normotensive-obese, young-adult African-American women, with and without parental histories of hypertension (PH+ comparison group, PH- control group, n=20). The upper panel compares the FMD before and after the exercise treatment. The lower panel compares the FMD after the exercise treatment.

Figure 1 presents the pre-exercise and postexercise differences in FMD. The upper panel shows that FMD increased significantly in both the control group without family histories of hypertension and in the comparison group with family histories of hypertension. The lower panel shows that FMD was less in the group with family histories of hypertension (mean ± 95% confidence intervals 11.3 ± 4.9% vs. 15.6 ± 4.9%, P<0.05) after the aerobic exercise treatment, but not before (see upper panel). FMD was positively correlated with heart rate, across parental histories and physical activity (r= 0.40, P<0.05).

## 6. Discussion

This is the first study to compare arterial stiffness and endothelial function in normotensive-obese young-adult AA women, with and without family histories of hypertension. The main finding of the study is that less flow-mediated vasodilation associated with reactive hyperemia was found in the women with family histories of hypertension 20 min after a single bout of aerobic exercise. Although the exercise increased flow-mediated dilation (FMD) in both study groups, we found no evidence of that there was less exercise-induced improvement in FMD in the group of women with hypertensive parents than in the control group of women with normotensive parents.

There were no significant differences in vascular markers of arterial stiffness (i.e., AI and pulse pressure). That there were no intergroup differences in arterial blood pressures and AI prior to exercise in this study is an indication that the study groups were well-matched for age, body weight, percentage of body fat, and aerobic capacity. Thus, as indicated by the experimental design, parental hypertension was the main unmatched variable testing the study's hypothesis.

Radial systolic, diastolic, and pulse pressures were measured and aortic pressures were computed, based on an estimate derived from the radial pressure waveform [32, 33]. Aortic pulse pressure is shown to be a reliable indicator of the relationship between left ventricular stroke volume and aortic compliance/elastance. Observed central (aortic) pulse pressure indicates heightened risk of adverse cardiovascular events such as ischemia/infarction. High aortic pulse pressure is also reported to be indicative of low aortic/arterial compliance or stiffness [36]. Measured more distally, brachial and radial pulse pressures are thought to be indicators of arterial augmentation of the propagated aortic pressure waves [37]. These pressure waves result from various causes including stiffness of the medium-sized and smaller peripheral arteries, only indirectly associated with adverse cardiovascular events [38]. AI is also a measure of arterial stiffness that is affected by various confounding factors such as shorter stature, lower heart rate and older age, which tend to increase it [39]. We confirmed the apparent physiological relationship between body stature and AI by virtue of our finding that body height and the baseline pre-exercise AI were negatively correlated. Our finding that parental history of hypertension was not predictive of increased arterial stiffness is not consistent with a report that parental hypertension was associated with increased aortic stiffness [40]. This inconsistency is likely explained by the facts that, in the aforementioned study the body weight, body mass index, systolic and pulse pressures were significantly greater in the group with parental histories of hypertension than in the group without such histories, whereas, these parameters were not different, well-matched between our study groups.

Although this is the first study demonstrating less FMD in normotensive-obese AA women, impaired FMD in nonobese AA normotensive subjects with parental histories of hypertension has been previously reported [41]. Exercise is shown to improve endothelial function in both healthy and unhealthy populations [42–44]. The findings of our study also show enhancement of postexercise FMD in both groups of women, with and without hypertensive parents. One of the more interesting aspects of our study is that a 30-min bout of aerobic exercise was required to unmask an apparent impairment in endothelial function, measured as less brachial FMD, in the women with parental histories of hypertension. This finding supports the study's hypothesis that the study subjects with hypertnesive parents have some degree of endothelial dysfunction despite having baseline resting arterial blood pressures in the normal range and brachial FMD not being different than their counterparts with normotensive parents.

FMD of the brachial artery is one, among several, measures of peripheral endothelial function. Such FMD is dependent on endothelium-derived nitric oxide, among other factors [45]. Marginally less ischemia-induced vasodilation during the periods of rest 20 minutes before, and significantly less after, a 30-minute bout of aerobic exercise was found in the women with hypertensive parents than in the women with normotensive parents. Such endothelial dysfunction is thought to reflect decreased bioavailability of nitric oxide and appears to involve higher-than-normal sodium [46] and fat [47] intake and features of diets across many cultures and ethnicities. It is plausible that our data could have been biased by high salt intake, especially if blood pressure responsiveness to salt is either a heritable trait or a behavior conditioned by dietary habits learned from parents. Although it is not clear whether sodium intake is a heritable trait across ethnicities, blood pressure responsiveness to high and low dietary salt intake is shown to exhibit significant heritability in a Chinese population consisting of 1,906 subjects [48]. A review of the putative mechanisms involved in this type of salt sensitivity is likely to vary with polymorphisms involving the renin-angiotensin system, aldosterone synthase, cytochrome p450 3A, epithelial sodium channel, sympathetic nervous system, *β*-3 subunit of G-protein, alpha-adducin, endothelial nitric oxide synthase, and kallikrein-kinin system genes [49]. It was beyond the scope of the present study to measure the sodium intake of the study subjects. However, our evidence for endothelial dysfunction in obese women with normal blood pressures should motivate the design of future studies on a larger group of normotensive-obese African-American women wherein salt intake and genotype are measured or controlled.

An epidemiological study involving 7,346 normotensive American subjects aged 25-74 years, without hypertension or cardiovascular disease reports that a wide pulse pressure is significantly associated with high risk of cardiovascular death, but only for the youngest subjects in whom cardiovascular disease was a very low risk [50]. The absence of significant effects on either central aortic or radial arterial pulse pressures, or on arterial augmentation index in the present study fails to support the hypothesis that stiffer arteries are more likely to be found in normotensive-obese AA female offspring of hypertensive parents than in those of normotensive parents. Because of the relatively small sample size of the present pilot study, this negative finding, as well as the aforementioned positive ones, should be interpreted cautiously pending a larger study.

## 7. Conclusion

A group of normotensive-obese young-adult African-American women with hypertensive parents seem to exhibit less capacity for ischemia-induced flow-mediated vasodilation after aerobic exercise than a matched control group with normotensive parents. These findings suggest that endothelial function in normotensive-obese, African-American women with hypertensive parents is more likely to be impaired only after a bout of aerobic exercise. We found no evidence that the endothelial function of the women with hypertensive parents is more resistant to improvement by aerobic exercise. Aerobic exercise and physical activity, in general, play a large role as a preventive strategy and therapeutic treatment for hypertension and cardiovascular disease [51, 52]. Longitudinal studies on similar cohorts should help determine whether endothelial function in normotensive-obese AA women predicts their development of hypertension and whether it can be improved enough by aerobic exercise to prevent their development of hypertension and cardiovascular disease.

## Figures and Tables

**Table 1:** Hemodynamic values before and after the exercise treatment.

Value	PH- (Control Group)		PH+ (Comparison Group)	
	Baseline	Exercise	Baseline	Exercise
AI (%)	5.5 ± 6.5	6.9 ± 4.1	8.7 ± 7.2	9.6 ± 8.2
HR (beats·min^−1^)	71.5 ± 10.3	79.5 ± 10.0	67.0 ± 4.7	75.2 ± 7.5
Brachial SBP (mm Hg)	112.5 ± 5.0	118.2 ± 8.0	113.3 ± 6.0	116.2 ± 6.5
Brachial DBP (mm Hg)	75.6 ± 4.2	76.2 ± 5.1	79.4 ± 4.6	80.2 ± 5.0
Brachial PP (mm Hg)	23.5 ± 2.5	25.7 ± 3.4	23.5 ± 2.8	24.5 ± 3.2
Aortic SBP (mm Hg)	99.5 ± 3.5	102.7 ± 6.8	104.5 ± 7.2	106.8 ± 7.8
Aortic DBP (mm Hg)	76.4 ± 4.8	77.8 ± 6.1	80.9 ± 5.4	81.7 ± 6.0
Aortic PP (mm Hg)	23.1 ± 4.1	24.9 ± 3.2	23.6 ± 3.4	25.1 ± 4.1

Values are means ± 95% confidence intervals.

PH- = women without parental histories of hypertension; PH+ = women with parental histories of hypertension; AI = augmentation index; HR = heart rate; SBP = systolic blood pressure; DBP = diastolic blood pressure; PP = pulse pressure.

## Data Availability

The data supporting this research article is available upon request to Profossor Vernon Bond, Jr., Director of the Exercise Science & Human Nutrition Laboratory, Howard University Cancer Center, Washington DC 20060, USA, email: vbond@howard.edu.
